# Mammalian ANP32A and ANP32B Proteins Drive Differential Polymerase Adaptations in Avian Influenza Virus

**DOI:** 10.1128/jvi.00213-23

**Published:** 2023-04-19

**Authors:** Thomas P. Peacock, Carol M. Sheppard, Margaret G. Lister, Ecco Staller, Rebecca Frise, Olivia C. Swann, Daniel H. Goldhill, Jason S. Long, Wendy S. Barclay

**Affiliations:** a Department of Infectious Disease, Imperial College London, London, United Kingdom; Lerner Research Institute, Cleveland Clinic

**Keywords:** influenza, ANP32, ANP32A, ANP32B, host factors, zoonotic, pandemic, equine influenza, canine influenza, avian influenza, swine influenza, pandemic influenza

## Abstract

ANP32 proteins, which act as influenza polymerase cofactors, vary between birds and mammals. In mammals, ANP32A and ANP32B have been reported to serve essential but redundant roles to support influenza polymerase activity. The well-known mammalian adaptation PB2-E627K enables influenza polymerase to use mammalian ANP32 proteins. However, some mammalian-adapted influenza viruses do not harbor this substitution. Here, we show that alternative PB2 adaptations, Q591R and D701N, also allow influenza polymerase to use mammalian ANP32 proteins, whereas other PB2 mutations, G158E, T271A, and D740N, increase polymerase activity in the presence of avian ANP32 proteins as well. Furthermore, PB2-E627K strongly favors use of mammalian ANP32B proteins, whereas D701N shows no such bias. Accordingly, PB2-E627K adaptation emerges in species with strong pro-viral ANP32B proteins, such as humans and mice, while D701N is more commonly seen in isolates from swine, dogs, and horses, where ANP32A proteins are the preferred cofactor. Using an experimental evolution approach, we show that the passage of viruses containing avian polymerases in human cells drove acquisition of PB2-E627K, but not in the absence of ANP32B. Finally, we show that the strong pro-viral support of ANP32B for PB2-E627K maps to the low-complexity acidic region (LCAR) tail of ANP32B.

**IMPORTANCE** Influenza viruses naturally reside in wild aquatic birds. However, the high mutation rate of influenza viruses allows them to rapidly and frequently adapt to new hosts, including mammals. Viruses that succeed in these zoonotic jumps pose a pandemic threat whereby the virus adapts sufficiently to efficiently transmit human-to-human. The influenza virus polymerase is central to viral replication and restriction of polymerase activity is a major barrier to species jumps. ANP32 proteins are essential for influenza polymerase activity. In this study, we describe how avian influenza viruses can adapt in several different ways to use mammalian ANP32 proteins. We further show that differences between mammalian ANP32 proteins can select different adaptive changes and are responsible for some of the typical mutations that arise in mammalian-adapted influenza polymerases. These different adaptive mutations may determine the relative zoonotic potential of influenza viruses and thus help assess their pandemic risk.

## INTRODUCTION

The natural host reservoir of influenza A viruses is wild aquatic birds. To efficiently replicate in mammalian hosts, avian influenza viruses need to overcome multiple barriers by adapting to several mammalian host factors (1). One major barrier to avian influenza virus replication in mammalian cells is the incompatibility of the viral polymerase with host acidic nuclear phosphoproteins of 32-kDa (ANP32) proteins (2). ANP32 proteins are essential for influenza genome replication (3, 4), and adaptation to the ANP32 proteins of a new host is generally the first and most frequent mutation seen during cross-species jumps (1, 5–7). Most avian species encode ANP32A proteins which are longer than their mammalian orthologues due to an exon duplication that results in a 33-amino acid insertion between the N-terminal leucine-rich repeat (LRR) domain and the C-terminal low complexity acidic region (LCAR). Viral RNA synthesis by avian influenza virus polymerase is supported by this longer avian-specific isoform of ANP32A, but not by the shorter mammalian form (2, 4).

Mutations in the heterotrimeric viral polymerase can enable efficient use of mammalian ANP32 proteins, of which PB2-E627K is the best characterized (2, 8, 9). However, some non-human mammalian influenza viruses do not contain PB2-E627K and have achieved mammalian adaptation through different mutations; for example, the swine-origin H1N1 2009 pandemic virus (pH1N1) has PB2 polymorphisms at positions 271, 590, and 591 which functionally compensate for the lack of E627K (10, 11). Furthermore, all equine influenza virus strains, Eurasian avian-like swine influenza viruses, and canine influenza viruses lack PB2-E627K, but contain an alternative adaptation, PB2-D701N, which was previously characterized as modulating mammalian importin binding (12, 13).

As well as differences in avian and mammalian ANP32 length, there are also differences in the level of redundancy to support influenza virus polymerase in different vertebrate hosts. ANP32A is the only ANP32 family member that efficiently supports influenza polymerase in birds, while in humans and most other mammalian influenza hosts ANP32A and ANP32B can both support polymerase activity to varying levels (5, 6, 14, 15). One exception is mice, in which only ANP32B can efficiently support influenza polymerase due to a polymorphism in murine ANP32A at position 130, a residue critical for the interaction between ANP32 proteins and viral polymerase (5, 6, 16). It has recently been shown that although both swine ANP32A and ANP32B can support mammalian-adapted polymerases, swine ANP32A has the more potent pro-viral function and can even partially support avian polymerases (15, 17). Similarly, in horses, dogs, seals, and bats, ANP32A appears to be a more potent pro-viral factor than ANP32B (15).

In this study, we aimed to understand whether alternative mammalian PB2 adaptations other than PB2-E627K function by adapting the polymerase to utilize mammalian ANP32 proteins, or whether they achieve adaptation through an ANP32-independent mechanism. We found that alongside PB2-E627K, -Q591R and -D701N specifically adapt avian polymerases to use mammalian ANP32 proteins. Furthermore, while PB2-D701N allows the polymerase to efficiently use both ANP32A and B proteins, -E627K specifically favors the use of mammalian ANP32B proteins. To support this finding, we used bioinformatics to show that viruses adapting to hosts in which ANP32B is the more potent pro-viral factor, such as humans and mice, generally adapt via PB2-E627K; while viruses that emerge in pigs, horses, or dogs, hosts with potent pro-viral ANP32A proteins, more often gain PB2-D701N or -Q591R. Using an experimental evolution approach, we passaged a pair of viruses containing the polymerase from avian influenza viruses in human cells and observed the emergence of the PB2-E627K mutation. This adaptation was not seen during passage of the same avian virus in human cells lacking ANP32B (BKO). Finally, we mapped the difference in ANP32B preference of polymerases containing E627K to the ANP32B protein LCAR, the region that has recently been implied to directly interact with the 627 domain in the influenza polymerase/ANP32 co-structure (18, 19).

## RESULTS

### PB2-Q591R, -E627K, and -D701N specifically adapt avian influenza polymerases to mammalian ANP32 proteins.

**FIG 1:**
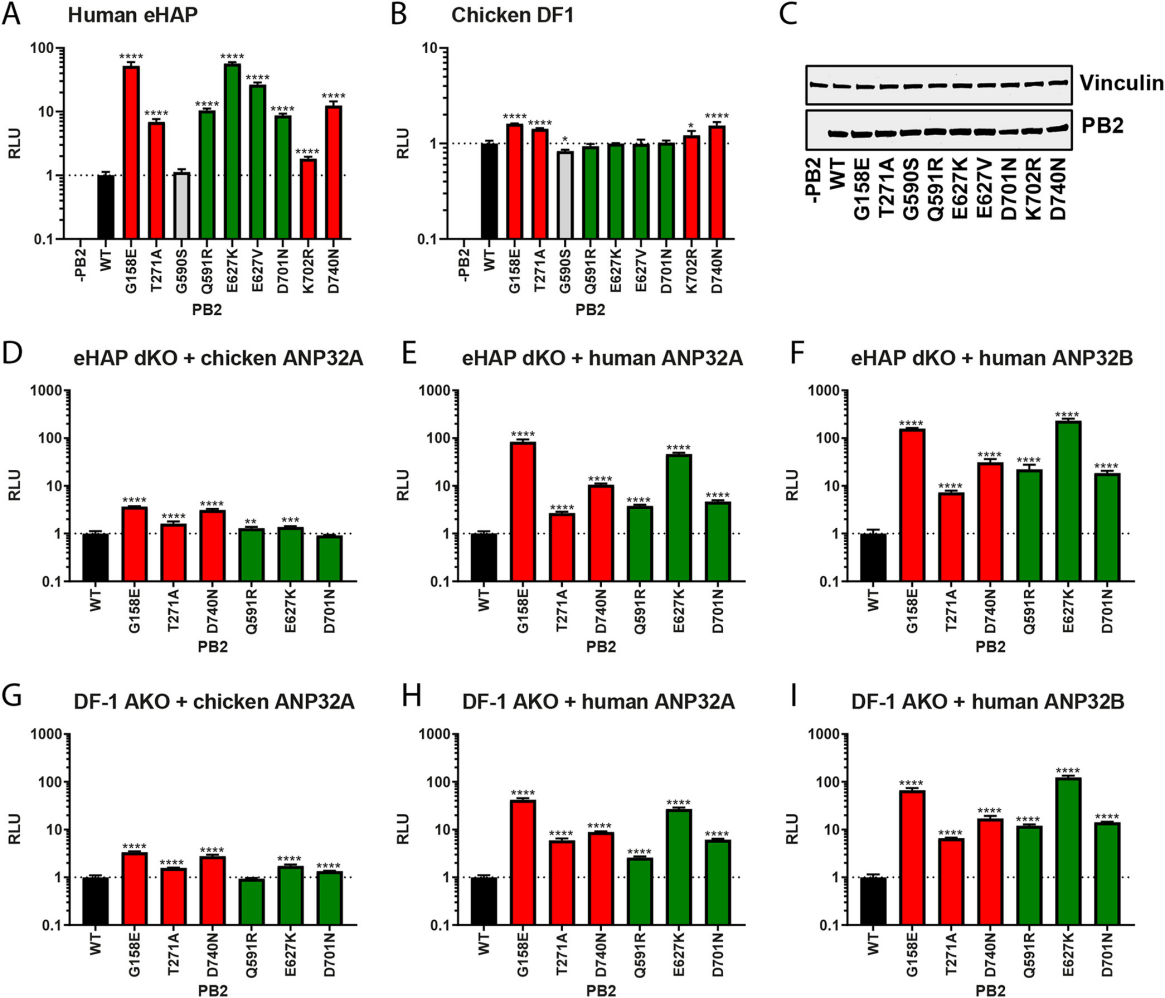
PB2 Q591R, E627K, and D701N specifically adapt influenza virus polymerase to human ANP32 proteins. Minigenome assays performed in wild-type (WT) human engineered-haploid (eHAP) cells (A) or WT chicken DF-1 cells (B) with avian 50-92 polymerase bearing different mammalian adaptations. (C) Western blot of mutant PB2 expression. Minigenome assays performed in human eHAP dKO cells (D to F) or chicken DF-1 AKO cells (G to I) with avian 50-92 polymerase bearing different mammalian adaptations transfected in along with different chicken or human ANP32 proteins. Data are representative of triplicate repeats (*n* = 3) and are plotted in triplicate. Data are plotted as means and standard deviation normalized to PB2 WT. Statistical significance was determined by one-way analysis of variance (ANOVA) with multiple comparisons against WT on log-transformed data. Log-normality of data were confirmed by Shapiro-Wilk test of normality. *, 0.05 ≥ *P* > 0.01; **, 0.01 ≥ *P* > 0.001; ***, 0.001 ≥ *P* > 0.0001; ****, *P* ≤ 0.0001.

We previously described a library of mammalian PB2 adaptations in an avian influenza virus polymerase backbone A/turkey/England/50-92/1991 (H5N1; 50-92) (20). Here, we expanded the library to include some additional mutants implicated in the literature as mammalian-adapting variants (Table 1). We compared the effect of each mutation on polymerase activity in wild-type (WT) human engineered-haploid cells (eHAP) (Fig. 1A) and chicken DF-1 cells (Fig. 1B). Consistent with our previous findings (20), the mutations displayed one of three phenotypes: (i) no significant increase in human or avian cells (G590S, gray bars), (ii) significantly increased polymerase activity in both human and avian cells (G158E, T271A, K702R, and D740N; red bars), or (iii) significantly increased polymerase activity only in human cells (Q591R, E627K/V, and D701N; green bars). This occurred despite equal expression of the PB2 WT and mutant proteins (Fig. 1C). This implies that the third set of PB2 mutants adapt the polymerase specifically in mammalian, not in avian, cells.

**TABLE 1:** PB2 mutants used in this study and effect shown by mutants with ANP32 proteins

Mutation	Group	Impact
G158E	2	Non species-specific polymerase activity boost
T271A	2	Non species-specific polymerase activity boost
G590S	1	Little or no effect alone
Q591R	3	Mammalian ANP32-specific adaptation (non-biased)
E627K	3	Mammalian ANP32-specific adaptation (ANP32B-biased)
E627V	3	Mammalian ANP32-specific adaptation (ANP32B-biased)
D701N	3	Mammalian ANP32-specific adaptation (non-biased)
K702R	2	Non species-specific polymerase activity boost
D740N	2	Non species-specific polymerase activity boost

A range of host factors have been implicated in the mammalian adaptation of avian influenza virus polymerase, including α-importins, DDX17, and ANP32 proteins (1). To investigate whether the PB2 mutations in our panel adapted polymerase to mammalian ANP32 proteins, we performed an ANP32 complementation assay in human cells lacking endogenous ANP32A and ANP32B (double knockout, dKO) (6, 14). We tested the complementation of polymerase activity for a subset of the group 2 and 3 PB2 mutants with ANP32 proteins from chicken or human (Fig. 1D to F).

As before the PB2 mutants displayed a differential phenotype which corresponded with that described in Fig. 1A and B. Group 2 mutants were boosted significantly more than WT PB2 by co-expression of either chicken or mammalian ANP32 (Fig. 1D to F, red bars); conversely, group 3 mutants were enhanced much more in the presence of human ANP32 proteins (Fig. 1D to F, green bars). This implies that the PB2 mutations at amino acids 591, 627, and 701 all enhance polymerase activity in human cells by specifically enabling efficient complementation by human ANP32 proteins.

We then considered whether any other host specific factors which differed between avian and mammalian cells might affect the complementation of influenza polymerase by ANP32 proteins. We reconstituted each polymerase containing different PB2 mutations in chicken DF-1 cells in which chANP32A expression was ablated by CRISPR editing (14), then rescued polymerase activity by co-expressing ANP32 proteins from chickens or human (Fig. 1G to I). Overall, the pattern of complementation in chicken cells was highly consistent with that seen in human cells, suggesting that mammalian-adapting polymerase mutations in PB2 enable mammalian ANP32 proteins to support polymerase activity even in chicken cells, and no other host factors which differ between avian and mammalian species are required for this phenotype. In both human and chicken cells lacking ANP32 proteins and complemented with chicken ANP32A, the group 3 PB2 mutations Q591R, E627K, and D701N did give a small but sometimes significant boost to polymerase activity; however, this boost was far less than that seen for the group 2 mutants (Fig. 1D to I).

These results indicate that PB2 Q591R or D701N, but not the other substitutions tested here, act in a similar manner to E627K and specifically enable the viral polymerase to utilize human ANP32A or -B proteins as a cofactor.

### ANP32 proteins across different species vary in their ability to support influenza polymerase activity.

**FIG 2:**
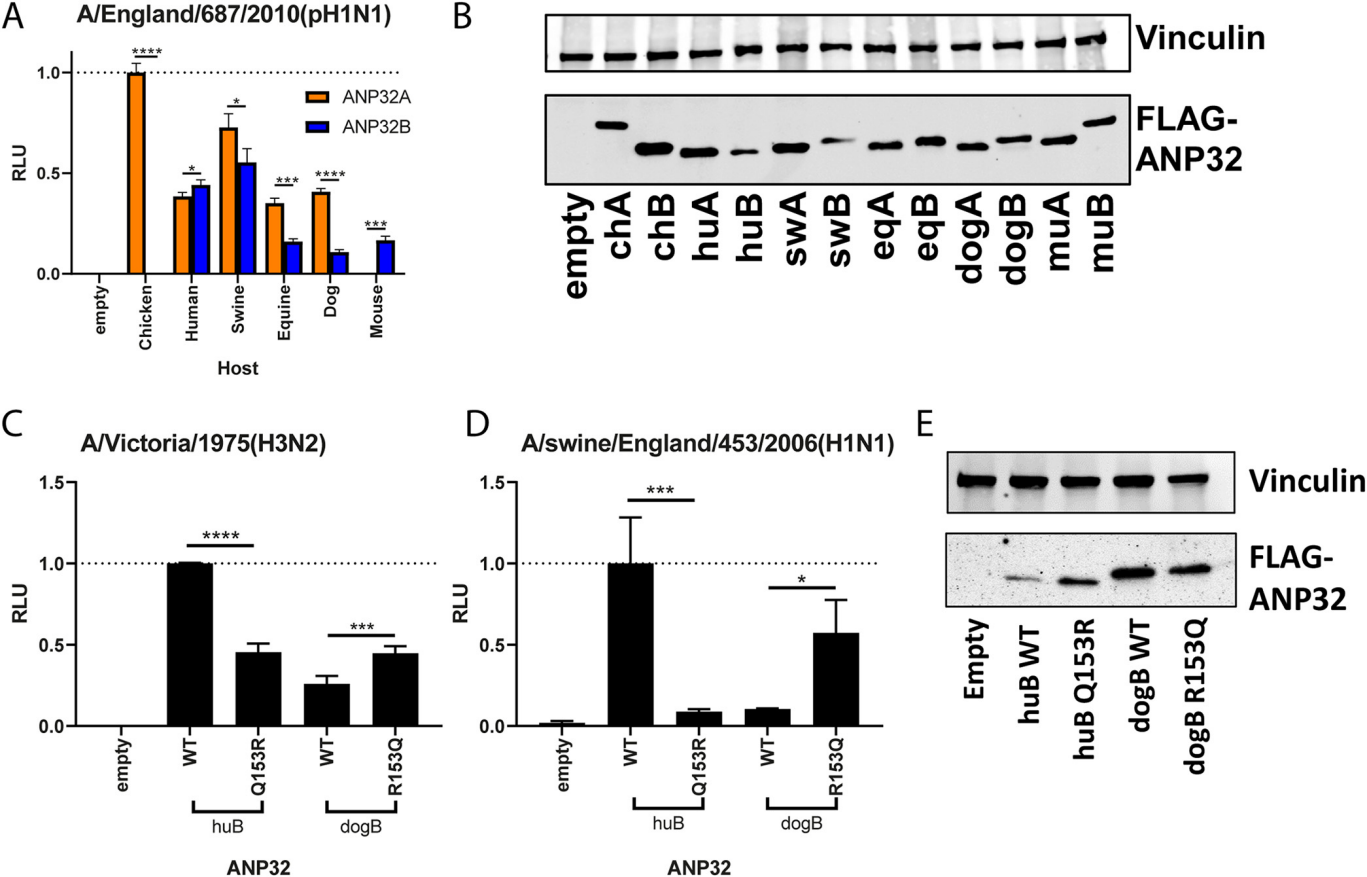
Different species’ ANP32 proteins display different patterns of dominance for influenza polymerase. (A) Minigenome assays performed in human eHAP dKO cells with a typical human influenza polymerase (A/England/687/2010 [pH1N1]), co-transfected with different mammalian ANP32 proteins expressed. Statistical significance was determined by multiple *t* tests between different species’ ANP32A and ANP32B proteins. (B) Western blot of ANP32 proteins as shown in panel A. (C and D) Minigenome assays performed in human eHAP dKO cells with different human or dog ANP32B mutants. Data are representative of triplicate repeats (*n* = 3) and are plotted in triplicate. Data plotted as means and standard deviation. Statistical significance was determined by one-way ANOVA with multiple comparisons, statistical tests performed between WT and mutant ANP32 proteins. *, 0.05 ≥ *P* > 0.01; **, 0.01 ≥ *P* > 0.001; ***, 0.001 ≥ *P* > 0.0001; ****, *P* ≤ 0.0001. (E) Western blot analysis of mutant ANP32 proteins as shown in panel C and D.

We next tested the ability of ANP32A and ANP32B proteins from different mammalian species relevant to influenza ecology to support influenza A polymerase activity. First, using a polymerase constellation from the 2010 pandemic H1N1 virus (A/England/687/2010) that is adapted to mammals via the PB2 mutation Q591R, we found that there was variation across species in the relative potencies of ANP32A and ANP32B. For example, in humans and mice, ANP32B was the more potent polymerase cofactor, while the ANP32A proteins of pigs, horses, dogs, seals, and bats more efficiently supported pandemic H1N1 polymerase activity (Fig. 2A,B) (5, 15). These subtle variations could not be accounted for by differences in protein expression (Fig. 2B), but instead were likely due to polymorphisms between the ANP32 orthologues in different species. For example, we and others have previously shown that the potent pro-viral activity of swine ANP32A is due to polymorphisms at positions 106 and 156, while the poor activity of mouse ANP32A and chicken ANP32B are due to polymorphisms at positions 129 or 130 (5, 6, 14, 15, 17). Using two additional different polymerase constellations—A/Victoria/75(H3N2), which harbors the adaptive PB2 mutation E627K, and A/swine/England/453/2006, which harbors the PB2 D701N mutation—we mapped the weak pro-viral activity of canine ANP32B to residue 153, which is glutamine in the human ANP32B but arginine in the canine orthologue (Fig. 2C to E).

### PB2 E627K, but not D701N, adapts influenza virus polymerase for preferential complementation by mammalian ANP32B.

**FIG 3:**
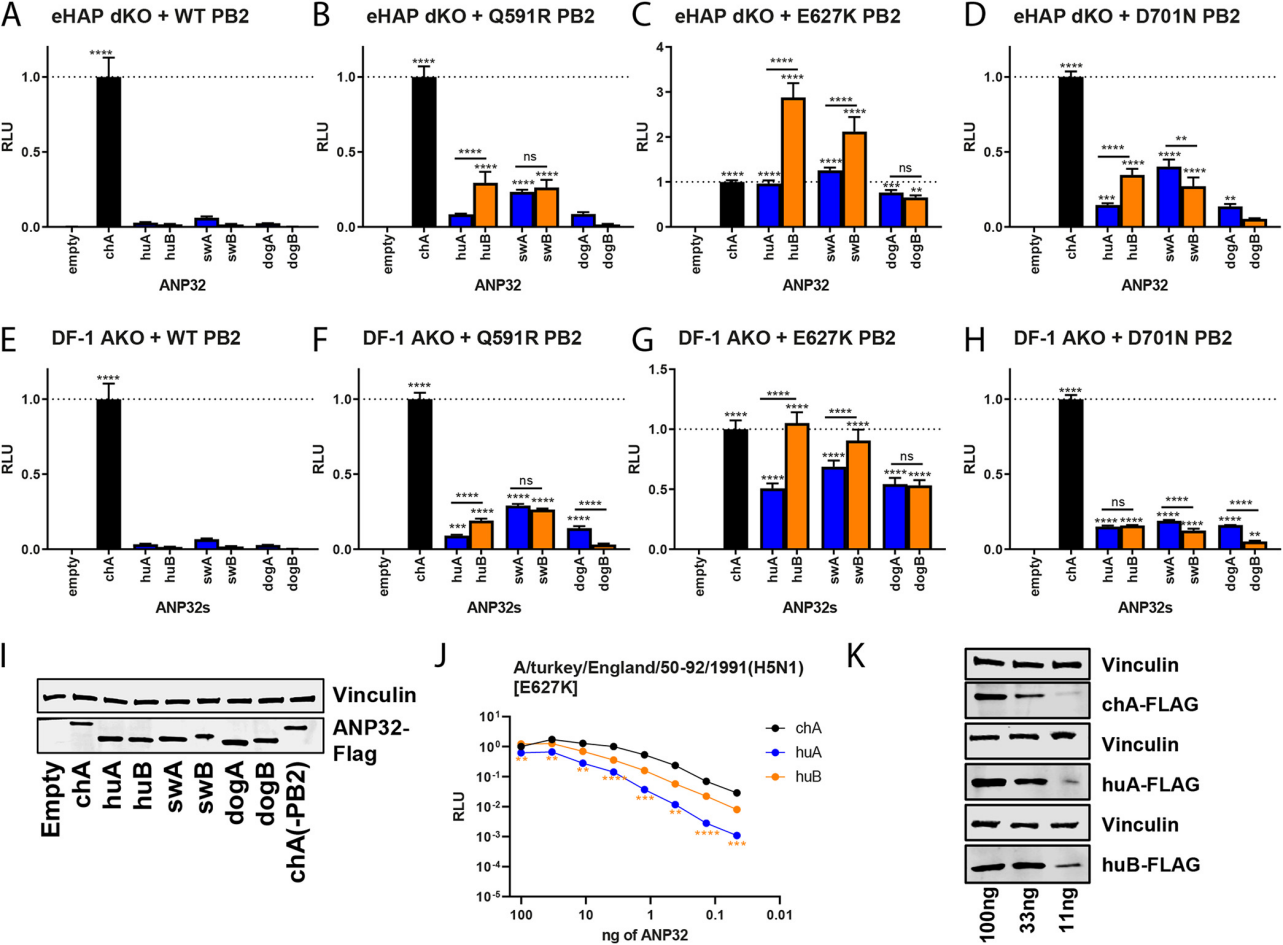
PB2 E627K shows a greater preference than Q591R or D701N for using mammalian ANP32B proteins. Minigenome assays performed in human eHAP dKO cells (A to D) or avian DF-1 AKO cells (E to H) with avian 50-92 polymerase bearing different mammalian adaptations transfected in along with different avian or mammalian ANP32A (blue bars) or ANP32B proteins (orange bars). Statistical significance was determined by one-way ANOVA with multiple comparisons, statistical tests without comparison bars indicate a comparison against empty vector and between ANP32A and ANP32B proteins from the same species. *, 0.05 ≥ *P* > 0.01; **, 0.01 ≥ *P* > 0.001; ***, 0.001 ≥ *P* > 0.0001; ****, *P* ≤ 0.0001. (I) Western blot of different ANP32 proteins used in panels A to H. (J) Minigenome assays performed in human eHAP dKO cells with chicken ANP32A, or human ANP32A or ANP32B, titrated in. Data are representative of triplicate repeats (*n* = 3) and are plotted in triplicate. Panels A to H replotted from same representative repeat as in Fig. 1D to I. Data plotted throughout as means and standard deviation. Statistical significance was determined by multiple *t* tests between human ANP32A and ANP32B, statistical tests performed between WT and mutant ANP32 proteins. *, 0.05 ≥ *P* > 0.01; **, 0.01 ≥ *P* > 0.001; ***, 0.001 ≥ *P* > 0.0001; ****, *P* ≤ 0.0001. (K) Western blot of highest concentrations used in ANP32 titration as shown in panel J.

To further investigate the compatibility between different polymerase constellations and different mammalian ANP32 proteins, we tested polymerase reconstituted with PB2 mutants from group 3 to see if they displayed any bias in ANP32 paralogue usage in human dKO or avian AKO cells (Fig. 3A to I). The relative efficacy of each ANP32 protein to support polymerase varied depending on the nature of adaptive mutation in PB2. For polymerase bearing PB2-D701N, in both human and chicken cells, human ANP32B was equal or superior to human ANP32A, whereas swine ANP32A was significantly more supportive than swine ANP32B and canine ANP32B was consistently poorly supportive (Fig. 3D and H). In contrast, for polymerase bearing E627K, both human and swine ANP32B were more potent than their respective ANP32A counterparts (Fig. 3C and G). Moreover, E627K polymerase was also somewhat supported by canine ANP32B, which is very poorly used by most polymerases (Fig. 3C and G; Fig. 2C and D) (15). PB2-E627K was more highly supported by human ANP32B than by ANP32A over a wide range of plasmid concentrations (Fig. 3J and K). For polymerase bearing PB2 Q591R, the pattern was intermediate: little difference was seen in swine ANP32 preference, while human ANP32B was significantly preferred over ANP32A and dog ANP32B was ineffective as a pro-viral factor (Fig. 3B and F).

These data illustrate a bias of different PB2 polymerase adaptations for different mammalian ANP32A or B proteins, which is particularly evident for PB2-E627K and, to a lesser extent, -Q591R, which show a preference for ANP32B proteins.

### Species with strongly pro-viral ANP32B proteins have a greater tendency to gain PB2 E627K.

Although PB2 E627K is the key polymerase adaptation in human seasonal influenza virus and is commonly found in human zoonotic infections as well as in laboratory-adapted mouse-passaged avian influenza viruses, it is rarely found in viruses that have crossed from birds into swine, dogs, or horses (10, 21). Instead, viruses endemic in those species tend to harbor the other ANP32-specific mammalian adaptations, PB2 Q591R and D701N. We therefore hypothesized that in humans, PB2 E627K might evolve as a specific adaptation to the strongly pro-viral ANP32B. Conversely, for other mammalian species such as dogs and horses, the selective pressure exerted by ANP32B would be weaker and adaptation would likely occur by PB2 D701N.

**FIG 4:**
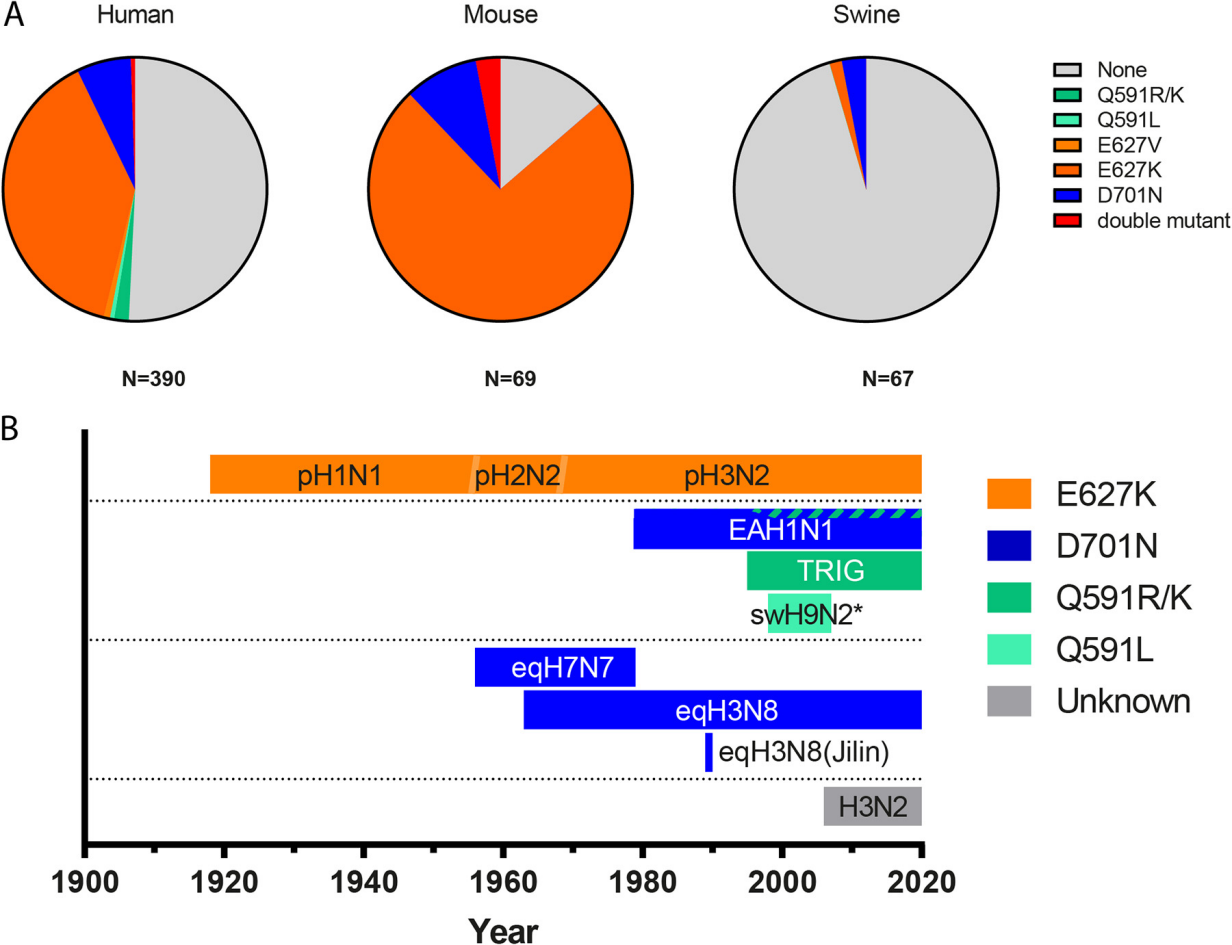
Mammalian species with dominantly pro-viral ANP32B are associated with E627K. Mammalian adaptations seen in avian-origin viruses during (A) zoonotic or likely dead-end cross-species infections/mouse passage experiments (middle panel only) and (B) stable circulation and prolonged adaptation to a mammalian host. Human and swine cross-species infections (panel A, left and right) were calculated by downloading all non-H1-3 human/swine influenza virus strain PB2s from NCBI, performing an alignment, curating out any seasonal human influenza segments, and looking at the identities of positions 591, 627, and 701. Mouse adaptation studies (panel A, middle) were calculated by performing a literature review of any influenza mouse adaptation studies using avian-origin influenza virus without any prior mammalian adaptation. Only virus strains with good evidence for having stably circulated in their respective mammalian species were included in the timeline (B). pH1N1/pH2N2/pH3N2 are represented by the same line because they contained the same PB2 originally from the spillover which caused the 1918 virus, reassorted into each new pandemic virus. Swine H9N2 (*) was included because phylogenetic and molecular evidence strongly suggested that although this virus appears to potentially co-circulate in both swine and chickens, it shows some clear mammalian adaptation markers.

To test this hypothesis, we first performed bioinformatics analysis on 526 viruses, comparing the proportions of viruses with mammalian adaptions found at sites 591, 627, and 701 during zoonotic infections, laboratory mouse adaptation studies, or sustained transmission of avian-origin PB2 segments in different mammalian species (Fig. 4A). PB2 E627K was strongly selected during human zoonotic events and mouse experimental adaptation, although other ANP32-specific adaptations, such as PB2-D701N, were also represented, albeit at a much lower proportion. Incursions of avian influenza viruses into pigs rarely showed adaptation at any of these three sites (Fig. 4A, right panel). This might be explained by the observation that swine ANP32A is somewhat supportive of non-adapted avian influenza virus polymerases (Fig. 3A and E) (15, 17). It is also noteworthy that the number of viruses exhibiting multiple ANP32-specific PB2 adaptations simultaneously is very low (double mutant), suggesting that these mutations are partially redundant.

In viruses that have crossed from birds and sustainably circulated in mammalian hosts, we saw a clear difference in PB2 adaptations in humans, compared to viruses endemic in swine, horses, and dogs. The only sustained avian-origin PB2 in humans (which transmitted during the 1918 Spanish influenza pandemic and was donated to subsequent pandemic viruses in 1957 and 1968) possesses E627K, whereas none of the polymerases from viruses adapted in other species, including pandemic 2009 H1N1, show this adaptation. Instead, such viruses contain D701N or Q591R/K/L; or, in the case of canine H3N2 viruses, none of the currently described mammalian ANP32-specific adaptations (Fig. 4B).

### Experimental evolution of an avian influenza virus in human cells shows that expression of ANP32B leads to PB2-E627K.

To investigate whether different ANP32 proteins drive different PB2 adapting mutations, we used an experimental evolution approach, serially passaging a virus with the polymerase gene segments derived from avian influenza viruses though human cell lines lacking either ANP32A (AKO) or ANP32B (BKO) (6).

**FIG 5:**
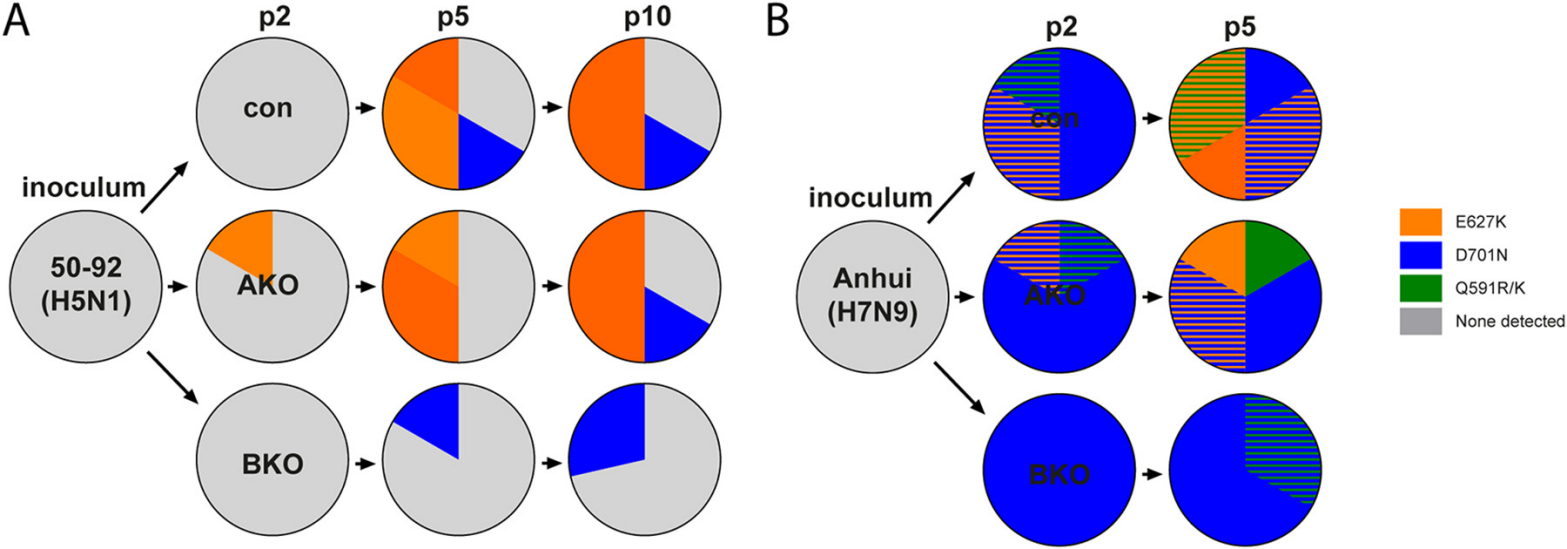
Experimental evolution of an avian influenza virus in human cells abrogated for ANP32B does not lead to the PB2 E627K adaptation. Sequencing summary of reassortant PR8 viruses containing the polymerases constellations from avian origin 50-92 (A) or Anhui (B) in human eHAP cells ablated for ANP32A (AKO) or ANP32B (BKO) or control cells (con). Each pie chart indicates 6 independently passaged populations. Depth of color indicates rough estimation of the proportion of the population: light shades indicate a mixed population of WT and indicated residues at a position, while darker shades indicate a complete change. Striped bars indicate a mix of both E627K and D701N. Gray slices indicate that no changes were detectible at positions 591, 627, or 701.

Six populations of a reassortant virus containing the Hemagglutinin (HA), Neuraminidase (NA) and Matrix (M) segments from the laboratory-adapted PR8 strain and the polymerase genes derived from avian influenza virus 50-92 (PB2-627E) were passaged 10 times through either control eHAP cells (which express both ANP32A and ANP32B) or cells that expressed human ANP32B only (AKO) or human ANP32A only (BKO). PB2 segments from each population were Sanger-sequenced at passages 2, 5, and 10. By passage 5, in both the control and AKO cells, three out of six (50%) populations had evolved PB2-E627K. This adaptation was not detected in the BKO cells, even by passage 10. Instead, in these cells, one virus population (17%) by passage 5 and two by passage 10 (33%), gained the D701N mutation (Fig. 5A). D701N was also seen in one or two populations in the WT and AKO cells by passage 10, respectively, while Q591R/K was not seen.

To confirm that this phenotype was not specific to a single strain of avian influenza, we repeated this experiment with a recombinant virus bearing the polymerase gene set from an H7N9 virus, A/Anhui/1/2013 (Anhui). The Anhui isolate had naturally gained PB2-E627K during zoonosis, but this was reverted to 627E by reverse genetics for the purpose of the passage experiment. In a similar manner to the H5N1 50-92 polymerase-bearing virus, the Anhui PB2 gained a mixture of E627K or D701N, as well as Q591K, in the control cells or AKO cells by passage 5; whereas in the BKO cells, every population evolved D701N or Q591K, but E627K was not detected (Fig. 5B).

Taken together, these observations suggest that the predominance for the PB2-E627K adaptive mutation seen in human cells is specifically driven by adaptation to utilize the strongly pro-viral host factor human ANP32B.

### The PB2-E627K preference for human ANP32B maps to the LCAR domain.

**FIG 6:**
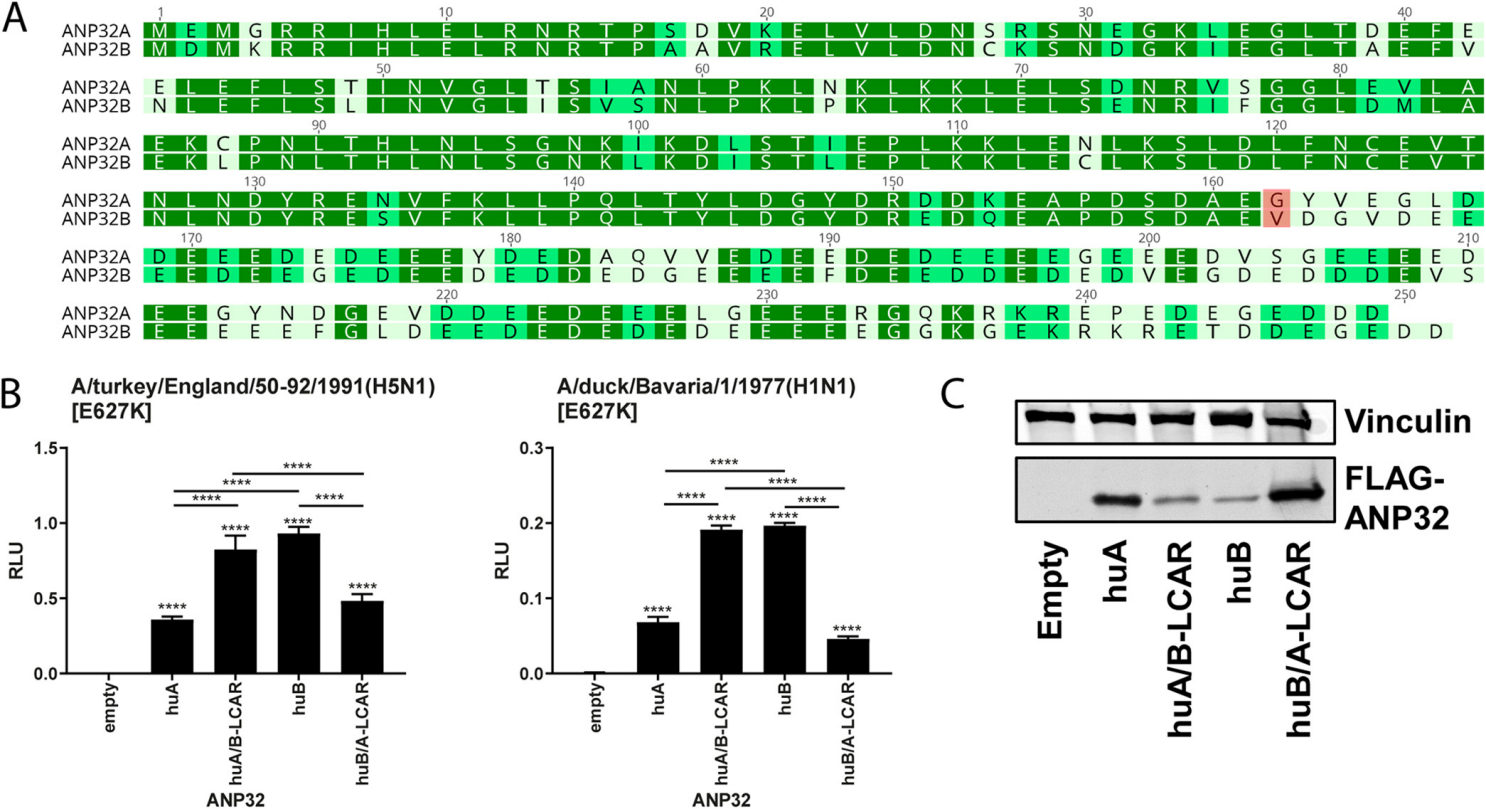
Differences in the low-complexity acidic region (LCAR) of human ANP32A and ANP32B are responsible for the preference of PB2 E627K viruses for ANP32B. (A) Non-aligned comparison of human ANP32A and ANP32B sequences, with sites of interest indicated in red. (B) Minigenome assays performed in human eHAP dKO cells with avian 50-92 or Bavaria polymerase with different mammalian adaptations transfected in along with different chimeric human ANP32 protein. Data are representative of triplicate repeats (*n* = 3) and are plotted in triplicate. Data are plotted as means and standard deviation. Statistical significance was determined by one-way ANOVA with multiple comparisons, statistical tests without comparison bars indicate a comparison against empty vector and between the different ANP32 proteins. ****, *P* ≤ 0.0001. (C) Western blot of chimeric human ANP32 proteins used in panel B.

To investigate the molecular basis of the superior ability of human ANP32B over ANP32A to complement two different avian influenza polymerase constellations bearing the mammalian-adapting PB2-E627K substitution, we generated human ANP32A/B chimeric ANP32 constructs. Because the LCAR is described as directly interacting with the 627 domain of PB2 (18, 19, 22), we switched the LCAR between human ANP32A and -B (from amino acid 161 to the C terminus; Fig. 6A, red highlight). Human ANP32A with the ANP32B LCAR was much more efficient at rescuing PB2-E627K polymerase activity than WT human ANP32A; while conversely, introducing the ANP32A LCAR onto human ANP32B reduced its capacity to support polymerase activity despite robust expression of the chimeric proteins (Fig. 6B, C). This pattern held true for both of the unrelated H1N1 and H5N1 avian-origin polymerases tested (Fig. 6B). This suggests that the functional preference for ANP32B shown by PB2-E627K polymerase maps to amino acid differences in the ANP32 LCAR domain.

## DISCUSSION

**FIG 7:**
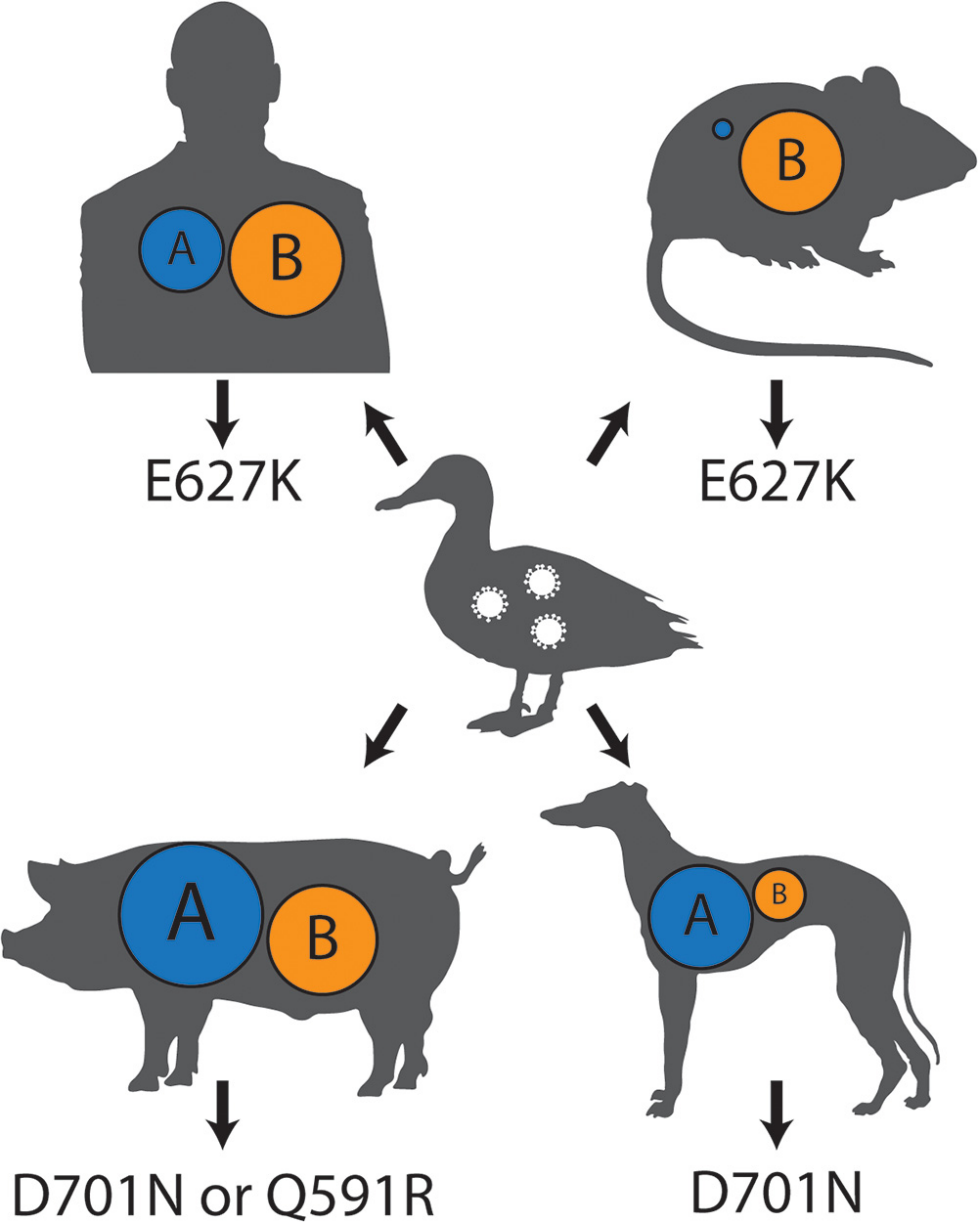
Model of how ANP32 protein dominance in different species may influence mammalian adaptation. Summary model of the data from this study showing how avian-origin influenza viruses adapt to the dominant pro-viral ANP32 protein in a new host and gain different adaptive mutations in PB2. Size of circles in each host indicate the relative pro-viral ability of its ANP32 proteins.

In this study, we showed that besides E627K, several other mutations in PB2 adapt the polymerase to utilize the shorter ANP32 proteins found in mammalian cells. However, PB2 E627K strongly biases polymerases toward reliance specifically on mammalian ANP32B. While ANP32A and ANP32B serve redundant pro-viral roles in many mammals, ANP32B is the dominant pro-viral factor in humans and mice, whereas in most other relevant mammalian hosts, such as pigs, horses, and dogs, ANP32A proteins is more potent (5, 6, 15, 17). This pattern shapes the adaptive evolution of avian viruses in these different mammalian hosts. Thus, adaptation in humans and mice tends to strongly select for PB2-E627K while pigs, dogs, and horses tend to select for PB2-D701N or Q591R/K. This was borne out in an experimental evolution study in which WT human cells and those lacking ANP32A drove viruses to gain PB2 E627K, whereas viruses passaged through cells lacking ANP32B did not acquire this mutation. Finally, we found that the strong preference for ANP32B proteins granted by PB2-E627K is due to differences between ANP32A and ANP32B sequence in the LCAR region of these proteins, a region shown to directly interact with the PB2 627 and nuclear localisation signal (NLS) domains (18, 19, 22). Overall, these data suggest that the evolutionary ecology of influenza virus polymerase varies in disparate species due to differences in mammalian ANP32 proteins (Fig. 7).

Previous studies have shown that varying expression of ANP32A isoforms resulting from differential splicing of transcripts in different avian species results in a different propensity to drive mammalian-like polymerase adaptations (e.g., PB2 E627K) (9, 23). Our work further expands on this concept that the pattern of ANP32 expression in a species can influence virus evolution. This finding may have implications for the relative risk of zoonotic or pandemic viruses emerging from different species; for example, although humans are often exposed to equine and canine hosts, there is a lack of evidence for zoonotic influenza infections originating from these species, although mismatched HA receptor-binding preference also likely contributes to this interspecies block (24).

Surprisingly, we found that PB2 D701N was an efficient adaptation to short mammalian ANP32 family members, regardless of whether the assay was performed in human or chicken cells. Previously, PB2 D701N has been described as an adaptation to a different set of host factors, the importin-α family (12, 13). Our results from avian cells would imply that importin-α adaptation is not the only phenotype of this mutation because, in the presence of chicken importins, polymerases reconstituted with this mutation were efficiently supported by mammalian ANP32 proteins.

Influenza virus polymerases co-opt a range of host factors for their replication and transcription (25). Our approach of using human and avian cells lacking pro-viral ANP32 family members has potential as a powerful screening method for investigating less well-defined mammalian ANP32 adaptations and discovering novel host factors that affect polymerase activity, including pro-viral and restriction factors, in human cells (26). Future work should further map the sequences in the ANP32B LCAR which direct the evolution of the PB2-E627K mutations and attempt to understand the preference for different PB2 adaptations driven by ANP32A or -B from a structural perspective.

## MATERIALS AND METHODS

### Cells.

Human engineered-haploid cells (eHAP; Horizon Discovery) without gene knockout (Control) or with ANP32A (AKO), ANP32B (BKO), or both knocked out (dKO) by CRISPR-Cas9, as described previously (6), were maintained in Iscove’s modified Dulbecco’s medium (IMDM; Thermo Fisher) supplemented with 10% fetal bovine serum (FBS; Biosera), 1% nonessential amino acids (NEAA; Gibco) and 1% penicillin-streptomycin (Pen-Strep; Invitrogen). Human embryonic kidney (293Ts, ATCC) and MDCK cells (ATCC) were maintained in Dulbecco’s modified Eagle medium (DMEM) supplemented with 10% FBS, 1% NEAA, and 1% pen-strep. Chicken fibroblasts (DF-1; ATCC) were maintained in DMEM supplemented with 10% FBS, 5% tryptose phosphate broth (Sigma), 1% NEAA, and 1% Pen-Strep. All mammalian cells were maintained at 37°C, 5% CO_2_ while DF-1 cells were maintained at 39°C, 5% CO_2_.

### Plasmid constructs.

The viruses and virus minigenome full strain names used through this study were A/duck/Bavaria/1/1977 (H1N1, Bavaria), A/turkey/England/50-92/1992 (H5N1, 50-92), A/Anhui/1/2013 (H7N9, Anhui), A/England/687/2010 (pH1N1), A/Victoria/1975 (H3N2), and A/swine/England/453/2006 (H1N1). Viral minigenome expression plasmids were either generated previously or created using overlap extension PCR (20, 27, 28). ANP32 expression constructs were made as previously described or generated using overlap extension PCR (2, 6, 15).

### Virus strains.

All virus work in this study was performed with A/turkey/England/50-92/1992 (H5N1; 50-92) or A/Anhui/1/2013 (H7N9) (K627E); reassortant viruses were generated by rescuing the polymerase, NP, and NS segments of the homologous virus with the HA, NA, and M segments of A/Puerto Rico/1/1934 (H1N1; PR8) as previously described (29). Virus was titered by plaque assay on MDCK cells. Virus PB2s were sequenced to confirm that no prior mammalian adaptation had been acquired during rescue or propagation. Infections were carried out at 37°C in relevant virus-containing serum-free medium (DMEM or IMDM, 1% NEAA, 1% P/S). At 1 h after infection, the medium was changed to serum-free medium supplemented with 1 μg/mL tosyl phenylalanyl chloromethyl ketone (TPCK)-treated trypsin (Worthington Biochemical). Passage experiments were performed by infecting cells at a multiplicity of infection (MOI) of 0.01; at 48 h after inoculation, viruses were harvested and prepared for the next passage.

### Minigenome assay.

eHAP dKO cells were transfected at around 50% confluence in 24-well plates using lipofectamine 3000 (Thermo Fisher) with the following mixture of plasmids: 100 ng of pCAGGs ANP32 or empty pCAGGs, 40 ng of pCAGGs PB2, 40 ng of pCAGGs PB1, 20 ng of pCAGGs PA, 80 ng of pCAGGs NP, 40 ng of pCAGGs *Renilla* luciferase, and 40 ng of polI vRNA-Firefly luciferase. DF-1 AKO cells were transfected in 12-well plates using double the amount of plasmid in eHAP cells, and polI vRNA-Firefly plasmids with a chicken polI site as described previously (30). At 24 h posttransfection, cells were lysed in passive lysis buffer (Promega) and luciferase bioluminescent signals were read on a FLUOstar Omega plate reader (BMG Labtech) using the Dual-Luciferase Reporter Assay System (Promega). Firefly signal was normalized to *Renilla* signal to give relative luminesce units (RLU). All assays were performed on a minimum of two separate occasions with the representative data shown.

### Western blotting.

To visualize protein expression during mini-genome assays, around 500,000 transfected cells were lysed in radioimmunoprecipitation assay (RIPA) buffer (150 mM NaCl, 1% NP-40, 0.5% sodium deoxycholate, 0.1% SDS, 50 mM Tris [pH 7.4]) supplemented with an EDTA-free protease inhibitor cocktail tablet (Roche).

Proteins were detected with mouse α-FLAG (F1804, Sigma), rabbit α-Vinculin (AB129002, Abcam), rabbit α-PB2 (GTX125926, GeneTex), and mouse α-NP ([C43] AB128193, Abcam). The following near-infrared (NIR) fluorescently tagged secondary antibodies were used: IRDye 680RD goat anti-rabbit (IgG) secondary antibody (AB216777, Abcam) and IRDye 800CW goat anti-mouse (IgG) secondary antibody (AB216772, Abcam). Western blots were visualized using an Odyssey Imaging System (LI-COR Biosciences).

### Experimental virus evolution.

At each passage, 1,000 PFU of avian influenza virus were inoculated in serum-free medium into confluent monolayers seeded in 6-well plates. After 1 h, medium was replaced with serum-free medium with 1 μg/mL of TPCK trypsin. At 48 h postinfection, the supernatant was harvested, spun down to remove cellular debris, and used for further passages. Samples were sequenced (where appropriate), frozen down, and stored at −80°C. Each passage experiment was done with 6 concurrent populations.

### Virus sequencing.

To sequence viruses, RNA was extracted from cell-free virus-containing supernatants using a viral RNA extraction minikit (Qiagen). cDNA synthesis was conducted using Superscript IV and the uni12-FluG primer (AGCGAAAGCAGG). Sequencing of PB2s was performed using two sets of primers with 5′-M13F or M13R primer sites (TGTAAAACGACGGCCAGTCCACTGTGGACCATATGGCC with CAGGAAACAGCTATGACCTGGAATATTCATCCACTCCC, and TGTAAAACGACGGCCAGTGGGAGTGGATGAATATTCCAG with CAGGAAACAGCTATGACCGCTGTCTGGCTGTCAGTAAGTATGC). PA was sequenced similarly (TGTAAAACGACGGCCAGTGCGACAATGCTTCAATCCAATG with CAGGAAACAGCTATGACCCTTCTCATACTTGCAATGTGCTC, and TGTAAAACGACGGCCAGTGGGCACTCGGTGAGAACATGGC with CAGGAAACAGCTATGACAACTATTTCAGTGCATGTG). PCR was performed using KOD Hot Start DNA Polymerase (Merck). PCR products were purified using the Monarch PCR and DNA Cleanup kit (New England Biolabs) and sequenced using the Sanger method with M13F or M13R primers.

### Bioinformatics analysis and literature review.

To assess the proportion of zoonotic influenza viruses with different mammalian ANP32 adaptations, the PB2 sequences of all non-H1, -H2, and -H3 human or swine influenza viruses were downloaded from the NCBI influenza virus database and aligned. Sequences which were determined to be of seasonal human influenza origin were identified by BLASTn and removed, and the proportions of viruses with adaptation at positions 591, 627, and 701 were calculated. For the mouse adaptation summary, we undertook an exhaustive literature search for any study taking an avian-origin virus without any prior mammalian adaptation and passaging it through mice in PubMed using the search terms “mouse,” “influenza,” and either “adaptation,” “adaption,” or “passage.” A list of the papers identified and included in this analysis is included in Table S1 in the supplemental material.

For the timeline of stably circulating avian-origin mammalian influenza virus strains, viruses were chosen due to the strength of the evidence that they came directly from birds into said species and not from another mammalian species—hence, pH1N1 swine-origin H1N1 and equine-origin canine H3N8 are excluded. Swine H9N2 was included because there remains fairly good evidence that although this virus may have continued to co-circulate between poultry and pigs, it does show several mammalian adaptations and therefore probably constitutes a swine-adapted avian-origin virus.

### Safety and biosafety.

All studies of infectious agents were conducted within biosafety level 2 facilities approved by the UK Health and Safety Executive and in accordance with local rules at Imperial College London.

### Data availability.

The manuscript contains no data deposited in external repositories.
